# Local fast-food environment, diet and blood pressure: the moderating role of mastery

**DOI:** 10.1007/s00394-018-1857-0

**Published:** 2018-11-13

**Authors:** J. D. Mackenbach, J. Lakerveld, E. Generaal, D. Gibson-Smith, B. W. J. H. Penninx, J. W. J. Beulens

**Affiliations:** 1 Department of Epidemiology and Biostatistics, Amsterdam Public Health Research Institute, VU University Medical Center, Amsterdam UMC, De Boelelaan 1089a, Amsterdam, 1081HV The Netherlands; 2 Department of Psychiatry, Amsterdam Public Health Research Institute, VU University Medical Center, Amsterdam UMC, Amsterdam, The Netherlands

**Keywords:** DASH diet, Fast-food restaurants, Food environment, Hypertension, Mastery

## Abstract

**Purpose:**

To examine the moderating role of mastery in the association of local fast-food restaurants (FFR) with diet quality and systolic blood pressure (SBP).

**Methods:**

We used cross-sectional data from 1543 adults participating in wave six of the Netherlands Study of Depression and Anxiety (NESDA). Data were collected between 2013 and 2016. Diet quality was defined by adherence with the dietary approaches to stop hypertension (DASH) diet. Individuals reported on their food consumption through a food frequency questionnaire and SBP was measured. Density of FFR in 1600 m, 800 m and 400 m circular buffers around the home postal code was calculated using Geographic Information Systems. We assessed the association between density of FFR, diet and SBP using linear regression analyses, testing for moderation by mastery.

**Results:**

Mean age was 52 years and 32.2% of the sample were men. Exposure to FFR ranged from 0 to 35 FFR per km^2^. Density of FFR was not significantly associated with DASH adherence or SBP. Only one out of the six interaction terms was significant, suggesting that for individuals with lower levels of mastery, higher density of FFR in an 800-m buffer was negatively associated with DASH adherence, while for individuals with higher levels of mastery, this association was positive.

**Conclusions:**

Exposure to FFR was not associated with diet quality and SBP, and we observed little evidence for moderation by level of mastery. This research question should be further explored in a large sample of healthy adults.

## Introduction

The consumption of a healthy diet is a priority for reducing obesity and risk of chronic diseases [1]. The dietary approaches to stop hypertension (DASH) diet has shown to effectively lower blood pressure without medication [2]. In addition, adherence to the DASH diet has been associated with lower risk of cardiovascular disease and all-cause mortality [3, 4]. It has been suggested that current food environments, characterized by high accessibility to high-energy and ultra-processed foods and an abundant availability of foods in general [5], form a barrier for the adherence to a healthy diet. There is some evidence that easy access to fast-food restaurants (FFR) is associated with unhealthier dietary consumption [6–8].

However, the evidence is inconsistent, which may be because some individuals are more sensitive to cues in the food environment than others [9]. For individuals living in environments where FFR are ubiquitous, reliance on perceived internal sources of control, such as sense of mastery, may provide the necessary resources to resist the cues from the (fast-)food environment [10]. Mastery (or ‘locus of control’) is a personality attribute that reflects the extent to which individuals perceive events to be under control [11]. This has been associated with a healthier diet [12–14], better cardiometabolic health and reduced risk for disease and death [15]. However, studies investigating the joint relations of environmental and psychological factors in relation to diet are scarce. The one study that investigated a similar research question concluded that mastery was only protective against metabolic risk in individuals living in environments with higher fast-food exposure [10].

The aim of the present study was to examine the moderating role of sense of mastery in the association of local FFR with adherence to the DASH diet and systolic blood pressure in participants of the Netherlands Study of Depression and Anxiety (NESDA). We hypothesized that sense of mastery and the density of FFR are independently and jointly associated with DASH adherence and systolic blood pressure, such that the strength of the relationship between the density of FFR and lower DASH adherence or higher blood pressure is greater in individuals with lower levels of mastery.

## Methods

### Study design

For the current cross-sectional study, we used data from the sixth wave of the NESDA study, which was collected across the Netherlands between 2013 and 2016. Recruitment for this study took place in the general population, in general practices (through a three-stage screening procedure), and in mental health organizations to recruit persons reflecting various settings and developmental stages of psychopathology. A total of 2069 individuals participated in wave six, of whom 396 were healthy controls, 568 had a current (6-month recency) diagnosis of depression (major depressive disorder or dysthymia) and/or anxiety (panic, social phobia, generalized anxiety disorder, agoraphobia) disorder, and 1105 individuals were at risk due to a (family) history of depressive and/or anxiety symptoms. Further details of this study can be found elsewhere [16].

### Exposure to fast-food restaurants

The primary measure of exposure to the food environment was density of FFR in a 1600-m Euclidean buffer around the home. We used data (collected in 2013) on the location of FFR from Locatus [17], a commercial company that performs yearly audits on all retailers in the Netherlands. We considered all fast food and take away outlets available in the Locatus database as FFR. In accordance with the classification of Lake et al. [18], fast food and take away outlets were defined as outlets serving hot food ordered and paid for at the till, foods cooked in bulk in advance and providing minimal table service (chain and non-chain FFR, take away and delivery outlets, grillrooms and kebab shops). We calculated the density of FFR in a 1600-m, 800-m and 400-m Euclidean buffer around the centroid of each individuals’ six-digit postcode. In the Netherlands, six-digit postcodes consist of approximately 17 residential addresses, as such comprising a relatively small area. In sensitivity analyses, we used (1) density of FFR as quintiles, rather than a continuous variable, and (2) the density of all types of food outlets in a 400-m, 800-m and 1600-m buffer. The latter was done to test whether the overabundant availability of food in general, rather than the density of FFR, is related to an unhealthier diet.

### Mastery

Individual sense of mastery was measured at wave six with an abbreviated version of the Pearlin Mastery Scale [11]. This abbreviated mastery scale has previously shown reasonable reliability (*α* = 0.67) in a non-institutionalized sample [19] and good reliability in individuals with a subclinical depression in the current study (*α* = 0.81) [20]. In the current study, internal consistency was excellent with *α* = 0.91. The respondents were asked to rate how much they agreed with five different statements on a five-point scale ranging from 1 (strongly disagree) to 5 (strongly agree). Negatively worded items were reversely coded prior to scoring, resulting in a score range 5–25, with higher scores indicating greater levels of mastery.

### Diet

Dietary intake was measured at wave six using a 238-item semi-quantitative Food Frequency Questionnaire (FFQ) with a reference period of 4 weeks [21]. This FFQ was based on an existing validated Dutch FFQ [22]. Overall diet quality was evaluated using an index of dietary adherence to the DASH diet, adapted from that of Fung et al [23]. The index consists of eight dietary components (grains/grain products; vegetables; fruits; low-fat/fat-free dairy; red and processed meat; nuts/seeds/dry beans; dietary sodium; and foods high in added sugar). Unfortunately, the FFQ did not allow for the assessment of dietary sodium intake. As such, the seven remaining components were divided into quintiles (separately for men and women), and the quintiles were summed to create an overall DASH scores ranging between 7 (minimal adherence) and 35 (maximal adherence).

### Blood pressure

Systolic and diastolic blood pressure was measured twice during supine rest, on the right arm, using an OMRON M4 IntelliSense digital blood pressure monitor (HEM-752A, Omron Healthcare, Inc., Bannockburn, Illinois, USA). We added 10 mmHg to the average of the two systolic blood pressure (SBP) measurements for those using antihypertensive medication (*N* = 364). These values represent the average decline in blood pressure in antihypertensive medication trials [24], and have been used in this manner before [25]. Analyses excluding those using antihypertensive medication generated similar results (data not shown).

### Covariates

Analyses were adjusted for socio-demographics, total energy intake, depression status and presence of food outlets other than FFR. The latter was done to correct for the fact that food outlets tend to co-locate. Given the high correlation between density of FFR and other food outlets, we derived the residuals of density of fast-food restaurants and all other restaurants and used this as predictor in the models. Models with SBP were additionally adjusted for current smoking status, body mass index, and alcohol consumption. As such, we present Model 1, which is adjusted for age, gender, marital status, years of education, household income, depression status and presence of food outlets other than FFR (for both DASH score and systolic blood pressure as outcome), and Model 2, which is adjusted for total energy intake (for DASH score as outcome) or for total energy intake, current smoking status, body mass index, and alcohol consumption (for systolic blood pressure as outcome). Because 95% of the NESDA participants had a North European ethnicity, we did not adjust for ethnic background. Given the mix of healthy, at-risk and ill individuals, we examined the difference in mastery between individuals with and without current depression using an independent *t* test. We further tested the interaction between depression status and the densities of FFR in 1600-m, 800-m and 400-m buffers in relation to DASH adherence and blood pressure, as well as the three-way interaction between FFR, mastery and depression status, which were non-significant in all models.

### Statistical analysis

From the 2069 individuals that participated in wave six, we excluded individuals who did not complete the FFQ (*N* = 433), who had extreme energy intake values [26] (*N* = 35) and those with missing data on their six-digit postcode (*N* = 58). The final analytical sample consisted of 1543 participants. Descriptives were presented across quintiles of DASH adherence and for the total sample. ANOVAs and Chi-square tests were conducted to examine differences in covariates across the DASH quintiles.

Missing values were handled using multiple imputations (*M* = 10) with the Predictive Mean Matching method, using all available variables [27].

The independent associations of density of FFR and mastery with adherence to the DASH diet and SBP were examined using linear regression analyses (quadratic and cubic transformations of the determinants were non-significant). For all statistical tests, a probability level of 95% was regarded as significant. The interaction between the independent variables was assessed by adding a cross-product term of the two continuous variables to the model. Following significant interaction terms, analyses were stratified by tertiles of mastery.

## Results

Characteristics of the analytical sample are presented in Table 1. Briefly, the mean (sd) age was 52.4 (12.9) years and 32.2% of the sample were men. Individuals with the highest DASH adherence (quintile 5) had the highest education level, consumed the lowest levels of alcohol and were least likely to be current smokers. The density of FFR in a 1600-m buffer ranged from 0 to 35, with a median of 1.6 and an interquartile range of 0.6–5.0. The density of all types of food outlets in a 1600-m buffer ranged from 0 to 243 restaurants per km^2^, with a median of 7.3 and an interquartile range of 3.2–22.6.

**Table 1:** Characteristics of individuals across quintiles (range) of dietary approaches to stop hypertension (DASH) adherence in NESDA (*N* = 1543)

Variable of interest	Q1 (8–16)	Q2 (17–19)	Q3 (20–21)	Q4 (22–24)	Q5 (25–34)	*p* value	Total sample
	*n* = 344	*n* = 256	*n* = 383	*n* = 213	*n* = 347		*n* = 1543
Age, years	51.1 (13.3)	52.2 (13.0)	52.7 (13.2)	53.0 (12.1)	53.3 (12.6)	0.24	52.4 (12.9)
Sex (% men)	31.4%	29.7%	31.3%	33.8%	34.9%	0.67	32.2%
Educational attainment, years	12.2 (3.2)	12.7 (3.3)	13.2 (3.2)	13.6 (3.4)	14.0 (3.1)	< 0.001	13.1 (3.3)
Household income (% > 3000€ net/month)	37.1%	38.4%	44.1%	53.6%	45.1%	0.41	43.1%
Marital status (% married)	48.0%	52.0%	53.5%	54.5%	46.7%	0.11	50.6%
Alcohol consumption, g/day	13.0 (19.9)	12.3 (17.3)	11.1 (14.7)	13.1 (15.4)	9.7 (12.4)	0.04	11.7 (16.1)
Energy intake, kcal/day	1997.6 (643.5)	2082.8 (622.9)	2083.2 (570.0)	2166.5 (561.2)	2312.3 (562.0)	< 0.001	2127.1 (602.8)
Smoking (% current smoker)	38.4%	23.0%	21.1%	17.4%	13.0%	< 0.001	22.9%
Body mass index, kg/m^2a^	26.7 (4.8)	27.0 (5.3)	26.7 (5.0)	26.1 (4.7)	25.2 (4.0)	< 0.001	26.3 (4.8)
Sense of mastery	18.5 (4.9)	18.9 (4.4)	19.5 (4.3)	19.2 (4.4)	19.8 (4.5)	0.002	19.2 (4.5)
Density of fast-food restaurants in a 1600-m buffer	3.5 (5.0)	3.6 (5.4)	4.1 (5.9)	4.7 (7.0)	4.9 (6.2)	0.007	4.1 (5.9)
Density of all food outlets in a 1600-m buffer	19.0 (29.5)	20.1 (34.4)	22.4 (36.7)	26.6 (44.6)	27.0 (36.3)	0.02	22.9 (36.3)
Depression status (% with MDD and/or dysthymia in the last 6 months)	27.9%	25.0%	19.6%	23.0%	17.0%	0.07	22.2%
Systolic blood pressure, mm per mercury	139.9 (21.4)	139.4 (21.1)	139.7 (21.8)	137.6 (20.9)	137.1 (21.7)	0.42	138.8 (21.5)
Hypertension (%)^b^	17.6%	13.2%	15.5%	14.1%	12.6%	0.43	14.8%
OR (95% CI) for being hypertensive^c^	Reference	0.84 (0.45; 1.45)	0.97 (0.59; 1.58)	1.01 (0.57; 1.81)	0.93 (0.54; 1.61)	–	–

*MDD* major depressive disorder. Numbers are means (SD), percentages or odds ratios (95% CI). *Q1* quintile 1 (least DASH adherent). *Q5* quintile 5 (most DASH adherent). *p* values indicate differences between the DASH quintiles, as derived from ANOVAs and Chi-square tests

^a^Participants were measured barefoot and wore light clothing. Weight was measured to the nearest 200 g with a calibrated electronic scale (TANITA model BC-418 MA; Tanita, Tokyo, Japan). Height was assessed to the nearest 0.1 cm with a wall-mounted stadiometer (SECA 240; Seca, Birmingham, United Kingdom). Body mass index (BMI; in kg/m^2^) was calculated as weight divided by square height

^b^Defined as having a systolic blood pressure ≥ 140 and a diastolic blood pressure ≥ 90, or currently receiving pharmaceutical treatment for hypertension

^c^Odds ratio for being hypertensive compared to individuals in the first DASH quintile. Adjusted for age, sex, educational attainment, dietary energy intake, alcohol consumption, body mass index, smoking status and severity of depressive symptoms

Individuals with a current depression reported lower levels of mastery compared to individuals without a current depression (mean (sd) = 15.8 (4.5) vs. 20.2 (4.1), *F* = 9.1, *p* < 0.001), but the cross-product terms of FFR density and depression status were non-significant, so analyses were not stratified by depression status.

The density of FFR in a 1600-m, 800-m and 400-m buffer was not significantly associated with DASH adherence or systolic blood pressure in either of the models (Table 2). The association between sense of mastery and DASH adherence showed a trend towards significance (*B* = 0.05, 95% CI − 0.00; 0.11), but not for SBP (*B* = 0.03, 95% CI − 0.20; 0.27). Of the six interaction terms tested, we found only one statistically significant interaction term, namely between density of FFR in an 800-m buffer and mastery (*B* = 0.03, 95% CI 0.01; 0.05) in relation to DASH adherence. Analyses stratified by tertiles of mastery revealed non-significant associations between density of FFR in an 800-m buffer and DASH adherence that differed in direction between the subgroups. For individuals within the lowest tertile of mastery, an increase in FFR density was non-significantly associated with lower DASH adherence (*B* = − 0.12, 95% CI − 0.28; 0.04), while for individuals within the highest level of mastery, an increase in FFR was non-significantly associated with higher DASH adherence (*B* = 0.12, 95% CI − 0.02; 0.26).

**Table 2:** Independent and joint associations of density of fast-food restaurants with adherence to the DASH diet and systolic blood pressure in NESDA (*N* = 1543)

	Model 1	Model 2	Interaction effect
	*B* (95% CI)	*B* (95% CI)	*B* (95% CI)
DASH adherence			
Density of FFR (no./km^2^) in 1600-m buffer	0.12 (− 0.04; 0.27)	0.11 (− 0.05; 0.26)	− 0.01 (− 0.05; 0.02)
Density of FFR (no./km^2^) in 800-m buffer	0.02 (− 0.06; 0.11)	0.01 (− 0.07; 0.10)	**0.03** (0.01; 0.05)
Density of FFR (no./km^2^) in 400-m buffer	0.03 (− 0.02; 0.08)	0.03 (− 0.01; 0.08)	0.01 (− 0.00; 0.02)
Mastery	0.05 (− 0.00; 0.11)		–
Systolic blood pressure			
Density of FFR (no./km^2^) in 1600-m buffer	− 0.03 (− 0.70; 0.64)	0.01 (− 0.65; 0.67)	− 0.08 (− 0.23; 0.07)
Density of FFR (no./km^2^) in 800-m buffer	0.10 (− 0.25; 0.45)	0.12 (− 0.22; 0.46)	− 0.04 (− 0.12; 0.04)
Density of FFR (no./km^2^) in 400-m buffer	− 0.02 (− 0.22; 0.18)	0.00 (− 0.19; 0.19)	− 0.01 (− 0.05; 0.04)
Mastery	0.03 (− 0.20; 0.27)		–

Bold values indicate statistically significant coefficients. Coefficients are the residuals of density of fast-food restaurants and all other restaurants. Model 1 is adjusted with covariates for age, gender, marital status, education level, household income and depression status. Model 2 for DASH adherence is further adjusted for total energy intake. Model 2 for systolic blood pressure also included the following variables: total energy intake, smoking (yes/no), body mass index, and alcohol consumption

*FFR* fast-food restaurants

Sensitivity analyses with the density of all types of food outlets in 1600-m, 800-m and 400-m buffers as well as sensitivity analyses with quintiles of density of fast-food restaurants showed similar results for both DASH adherence and SBP outcomes (data not shown).

## Discussion

We hypothesized that some individuals may be more sensitive to cues in the food environment because they lack perceived internal sources of control, such as sense of mastery, while for others, a higher sense of mastery may provide them with the necessary resources to resist the cues in the food environment, such as those from fast-food restaurants. However, in this study of Dutch healthy controls, individuals at risk of depression or anxiety and individuals with a current depressive or anxiety disorder, we found little evidence of independent or joint effects of density of fast-food restaurants around the home, sense of mastery and adherence with the DASH diet or systolic blood pressure.

We could not confirm the hypothesis that density of FFR was associated with diet quality and systolic blood pressure. Although fast-food consumption is associated with lower diet quality [28, 29], the effect sizes of an association between exposure to FFR and fast-food consumption may have been too small to detect with an overall dietary outcome measure. Another explanation may be the characteristics of the sample under study, although it could be speculated that individuals at risk of, or currently suffering of anxiety and/or depressive disorders may be more vulnerable to an unhealthy food environment given the links between impulse control disorders, addictive disorders and anxiety and depressive disorders [30].

The independent association of mastery with DASH adherence showed a trend towards significance, and we observed significant interaction by mastery in the association between density of FFR in an 800-m buffer and DASH adherence. Stratified analyses suggested that the association between density of FFR and DASH adherence may differ between those with higher and lower levels of mastery, but the non-significant coefficients require a cautious interpretation. The independent association between mastery and diet quality would be in concordance with previous studies investigating this association [13–15]. Future studies could investigate whether domain-specific control beliefs such as self-efficacy with regard to healthy eating is a stronger moderator of food environment–diet associations than general locus of control.

The findings of this study must of course be interpreted within the constraints of its limitations. We used a sample of individuals with and without recent depressive or anxiety disorders; therefore, we may not have been able to fully account for the interplay between depressive disorders and sense of mastery. As such, the observed results may not be generalizable to the general Dutch population. In addition, women were overrepresented in this sample, it was a cross-sectional study and inherent to FFQs, absolute food intake may be under- or overestimated [22]. Third, we assumed that the presence of (fast-)food outlets in circular buffers around the home would be an accurate representation of exposure to individual food environments, but we had no information on where individuals went for their (fast-)food or how the food environment changed during the study period. This is a common issue in health geography issues, and using different buffer sizes we tested the robustness of our results. Finally, the database containing locations of food retailers was not validated. However, commercially available data are generally considered to be of good quality [31] and a previous study comparing the Locatus database with a neighbourhood audit showed discrepancies in only 5 out of 285 outlets under study [32], suggesting limited risk of exposure misclassification. Nonetheless, we were able to use a large sample of adults living across different areas in the Netherlands, we had objective data on blood pressure and a detailed database of the location of food retailers.

In conclusion, we could not confirm the hypothesis that exposure to fast-food restaurants around the home was more strongly related to poorer diet quality in individuals with lower levels of mastery. Both the independent and joint associations should be further examined in future studies conducted in the general population and with larger sample sizes.
